# Views of Swedish Elder Care Personnel on Ongoing Digital Transformation: Cross-Sectional Study

**DOI:** 10.2196/15450

**Published:** 2020-06-16

**Authors:** Katarina Baudin, Christine Gustafsson, Susanne Frennert

**Affiliations:** 1 School of Health, Care and Social Welfare Mälardalen University Eskilstuna Sweden; 2 Internet of Things and People Research Center Department of Computer Science and Media Technology Malmö University Malmö Sweden

**Keywords:** elder care, welfare technology, gender, municipality, participation, digitalization, age

## Abstract

**Background:**

Swedish municipalities are facing demographic challenges due to the growing number of older people and the resulting increased need for health care services. Welfare technologies are being launched as possible solutions for meeting some of these challenges.

**Objective:**

The aim of this study was to explore the perception, experimentation, evaluation, and procurement of welfare technology practices among professionals working in municipal elder care in relation to their gender, age, and profession.

**Methods:**

Data for this explorative cross-sectional study were collected from 393 responses to a web-based survey on municipal elder care in Sweden. Chi square tests were performed to determine the associations.

**Results:**

The results revealed gender, age, and professional differences in perspectives of municipal elder care workers. Differences were particularly evident in attitudes toward technology, both the use of technology in general and in the workplace, and involvement and participation in decision making regarding the procurement of new welfare technologies. Men (37/53, 70%) expressed a more positive attitude toward and curiosity regarding new technologies than women (157/336, 46.7%) (*P*=.03). Regarding age, the younger respondents (18-24 years old) perceived the digital transformation in the workplace as “too slow” (4/4, 100%), whereas the majority of older respondents (65-74 years old) perceived it as happening at the “right pace” (4/7, 57%). The elder care personnel felt encouraged by management to explore and experiment with new welfare technologies, but never did so either for management or with patients. Even though the majority of the respondents were women, more men (4/7, 57%) were involved in the procurement process for welfare technology devices and solutions than women (98/336, 29.2%) (*P*<.001).

**Conclusions:**

Personnel working within municipal elder care were generally very positive toward new technologies. However, both gender and age differences may influence these perspectives such as the personnel’s resistance to welfare technology and patients’ participation in welfare technology usage and deployment. Different levels of participation in the decision-making process regarding new technology deployment may negatively affect the overall digital transformation within municipal elder care.

## Introduction

### Background

Due to demographic changes, developed countries have growing concerns about the future challenges aging populations will present to their welfare systems [1-3]. In the Nordic countries, welfare technologies have been introduced as an important means of meeting these challenges [1]. In Sweden, the welfare system was developed to promote universal rights and social equality. Despite the increasing demand on the limited resources of welfare services, it is believed that welfare technology can help to sustain these rights through the digital transformation of care for all Swedish citizens, including older people in need [4]. The Swedish National Board of Health and Welfare believes that digital technology can help older individuals and people with disabilities to feel safe and to participate in society [5].

The expectation is that welfare technology will “gain time” for health care personnel to engage in human contact and that it will increase patients’ self-management and independence [6-8]. Societal changes and scarce resources are constant challenges to both welfare technology practitioners and policy makers whose aim is to reap the benefits of technological change for individuals and for society as a whole [9-12]. Municipalities and county councils or regions in Sweden provide welfare technology as aids and housing adaptations. Examples of welfare technologies provided at the municipal level are social security alarms that can be used to call for emergency help, electronic home services that replace or supplement physical visits with digital contacts, advanced toilets with flush and drying functions, key-free home services that replace physical keys with digital key management systems, individual rehabilitation training through game consoles in the home, camera surveillance during the night, and medicine reminders of when to take prescriptions, which may be linked to an alarm system [13]. Welfare technology designed to support daily living must be understood and assessed in their context and from a social perspective [1,13,14]. The provision of a good lifestyle through welfare technology is affected by decisions made at the municipal level; it is therefore important to understand how the decisions are made at the municipal level when new welfare technologies are purchased.

Exploring the role of welfare technology in municipal elder care is important because the use of technology in home care and in health care is constantly increasing [12,14-21]. However, research on the use of welfare technology in elder care has often focused on the attributes of leadership and management. A review of welfare technology use in elder care highlighted that the success factors in integrating welfare technology into elder care are having clear goals, incentives, and strong leadership; an infrastructure with proper capacity; a well-functioning organizational structure; collaboration with others; and awareness of resources [14]. However, the review did not identify success factors relating to gender, age, or work experience among the various care professionals involved in welfare technology work and in decision making regarding welfare technology.

### Aim

The aim of this study was to explore the perceptions of professionals involved in municipal elder care regarding the use of welfare technology and to assess the influence of gender, age, and profession on their perceptions, experimentation, evaluation, and procurement of these technologies.

## Methods

### Survey Design

This explorative cross-sectional study involved a web survey and a descriptive data analysis. In May 2018, the authors sent a hyperlink to a web-based survey to all municipal registrars in Sweden (N=290). The registrars were asked to distribute the link to those working with and involved in welfare technology work in elder care organizations. In the information letter, the researchers stated that that the respondents’ answers would be confidential and not linked to any individual. Participation was voluntary. Anonymity was assured and participants were informed of the survey aim.

To explore the aim of the study, in the absence of any previous research addressing this topic, a new questionnaire was designed, and its psychometric properties were tested for both validity and reliability [22]. The survey covered five question areas with a fixed number of options (3-6 possible answers) for the respondent to choose from. The areas included questions on perceptions of the speed of technological change, participation in decision making in relation to welfare technology, experimentation and exploration of welfare technology at work, involvement in procurement, and “about you.” The survey also included four areas of open-ended questions addressing the concept, advantages and potential, barriers to use, and evaluation methods for welfare technology. The results of the open-ended questions have been published in a separate article [23]. The questionnaire was pilot-tested with three potential users, and its content, face validity, and test-retest reliability were examined prior to distribution [24]. Four established and experienced researchers rated the content validity of the items using a 4-point scale (4=very relevant, 3=quite relevant, 2=somewhat relevant, 1=not relevant). The same experts were asked to determine the face validity by answering the following question: “Please write your reflections and comments about the questionnaire concerning readability, clarity, and layout.” After 3 weeks, their comments on the content and face validity were reviewed by the authors and discussed [24], and some changes were made to the questionnaire. The test-retest reliability tests, conducted at 2-week intervals with three potential users, showed that the questionnaire was easy to understand and answer and took 10-15 minutes to complete [25]. The focus of the present study is to analyze the quantitative data from the fixed-option questions (see Multimedia Appendix 1) in the questionnaire.

### Sample

In this explorative cross-sectional study [22], the respondents were personnel who worked with welfare technology in municipal elder care. Table 1 summarizes the respondents’ professional affiliations.

**Table 1:** Characteristics of the study population (N=393).

Characteristic		n (%)
**Sex**		
	Male	53 (13.5)
	Female	336 (85.5)
	Missing	4 (1.0)
**Age (years)**		
	18-24	4 (1.0)
	25-34	43 (10.9)
	35-44	75 (19.1)
	45-54	146 (37.2)
	55-64	118 (30.0)
	65-74	7 (1.8)
**Working experience**		
	Less than 1 year	18 (4.6)
	1-4 years	52 (13.2)
	5-9 years	58 (14.8)
	10-19 years	125 (31.8)
	More than 20 years	140 (35.6)
**Profession/Roles**		
	Information technology staff	29 (7.4)
	Chief medical nurse responsible	78 (19.8)
	Chief rehabilitation office responsible	28 (7.1)
	Occupational therapist/physiotherapist	103 (26.2)
	Specialist dementia nurse	51 (13.0)
	Other (manager/electronic health strategist)	104 (26.5)

### Statistical Analysis

Descriptive statistics were used to summarize the characteristics of the sample and its subgroups. Crosstabulation with chi square computation was used for analysis of categorical variables, which is a nonparametric test recommended when analyzing ordered categorical data [22].

The associations between the variables are summarized in Multimedia Appendix 2. The occupational therapist (OT) and physiotherapist (PT) respondents who have direct contact with patients in their everyday work were grouped into the category “OT/PT.” Managers and electronic health (eHealth) strategists who work at the strategic level in elder care organizations and do not have daily contact with patients were grouped as “other.” The survey data were imported into and analyzed with IBM SPSS version 24.

### Ethics

This study did not include any personal or sensitive information that required ethical approval under the standards of the Swedish Research Council [26]. The study followed the guidelines for research ethics issued by the Swedish Research Council [26].

## Results

### Respondents’ Characteristics

The online survey elicited 393 responses. Table 1 presents the respondents’ demographic data.

### Perceptions of the Speed of Change

The perceptions of personnel in elder care regarding the speed of digital transformation in their organizations was investigated through a question asking the participants to “grade the speed of the digital transformation in your workplace” (see questionnaire, Multimedia Appendix 1). The majority of both women (245/336, 72.9%) and men (46/53, 87%) answered that it was “too slow,” and the chi square test showed a significant association of gender (*P*=.05). Regarding age, all of the younger respondents (18-24 years old) perceived the speed of digital transformation as “too slow” (4/4, 100%), whereas the majority of older respondents (65-74 years) perceived the digital transformation in the workplace as happening at the “right pace” (4/7, 57%).

The perceptions of surveyed personnel regarding the overall speed of technological change in health care was also examined (Multimedia Appendix 1: question 2), demonstrating an association between gender and attitudes toward technology and digitalization within health care organizations (*P*=.03). Although 157/336 (46.7%) of the women disagreed with the statement that the change in health care organizations was too rapid, 37/53 (70%) of the men also disagreed with the statement. The results also showed an association between the respondents’ profession/role in the organization and their perception as to whether technology and digitalization have made health care organizations change too quickly (*P*=.04). Specifically, 21/29 (72%) of the information technology (IT) staff, 20/28 (71%) of the chief rehabilitation officers responsible, and 42/78 (54%) of the chief medical nurses responsible disagreed with the statement that technology and digitalization have made health care organizations change too quickly. These results indicate that in their experience, technological developments in elder care have not been occurring fast enough.

The questionnaire also addressed whether the personnel perceived their workplaces as using welfare technology optimally (Multimedia Appendix 1, question 3). The chi square test showed no effect of gender but a strong association with profession/role (*P*<.001). Approximately half of the IT staff (13/29, 45%), chief medical nurses responsible (35/78, 45%), and OT/PTs (53/103, 51.4%) completely disagreed with the statement that their workplace optimized welfare technology.

To learn more about the respondents’ attitudes and perceptions of technology (Multimedia Appendix 1, question 4), the respondents indicated their level of interest in technology. Here, some gender and age differences emerged. Overall, 191/336 (49.1%) of the women responded that “they use technology when most people do,” whereas 30/53 (57%) of the men answered that they “like new technology and use technological solutions before most people.”

The distribution of responses showed that most 18-24-year-old respondents (3/4, 75%) answered “I like new technology and use technological solutions before most people,” whereas none of the 65-74-year-old respondents answered that they “love new technology.” Considering profession/role, 16/29 (55%) of IT personnel, 33/78 (42%) of chief responsible rehabilitation specialists, and 49/103 (47.5%) of those in the “others” category responded that they “like new technology and use technological solutions before most people.” In the groups of specialist dementia nurses (36/51, 71%), OT/PTs (53/103, 51.4%), and chief responsible rehabilitation specialists (15/28, 54%), the majority answered that “they use new technology when most people do.” 

### Encouragement, Exploration, and Experimentation With Welfare Technology

The next question examined how the personnel perceived the importance to their management or closest superior of their working with welfare technology (Multimedia Appendix 1, question 5). The chi square test results showed no effect of gender and age but a strong association between profession/role and encouragement from management to use welfare technology (*P*=.01). Most respondents working in IT (26/29, 90%) reported that they were encouraged by their managers to test and use welfare technology, as did the “other” group of professionals such as eHealth strategists and unit managers (82/103, 79.6%). The results showed almost the same pattern among the other professionals, including the chief medical nurses (yes: 57/78, 73%; no: 21/78, 27%) and chief responsible rehabilitation specialists (yes: 22/28, 79%; no: 6/28, 21%).

The questionnaire then asked how much the respondents experimented with and explored welfare technology in their everyday work (Multimedia Appendix 1, question 6). The chi square test results showed an association between gender and experimentation with new welfare technology (*P*<.001). Over half of the women (186/335, 55.5%) answered that they do “not very often” experiment with welfare technology, whereas among the male respondents, only 19/53 (36%) answered that they “do not very often” experiment. The chi square test showed an independence between age and experimentation of welfare technology in daily work. There were associations between experimentation with welfare technology and the respondents’ profession/role (*P*=.02). The majority of the personnel responded that they never explored or experimented with new welfare technology solutions (IT staff: 12/29, 41%; chief responsible medical nurses: 46/78, 59%; chief responsible rehabilitation specialists: 14/28, 50%; OT/PTs: 31/102, 30.3%; specialist dementia nurses: 36/51, 71%; “others”: 45/103, 43.7%).

Two questions assessed whether the respondents explored and experimented with new welfare technology solutions with management and clients/patients in their everyday work (see Multimedia Appendix 1, questions 7 and 8). The results showed that more men than women experimented and explored welfare technology with management. Neither age nor work experience had any association with opportunities to experiment and explore welfare technology with management.

The chi square test also showed an association of profession/role with experimentation and exploring welfare technology with management. The IT staff reported that they occasionally (10/29, 35 %) or never (11/29, 38%) experimented and explored welfare technology with management. Among the chief responsible medical nurses, 35/78 (45%) chose the “never” option. The majority of OT/PTs and specialist dementia nurses gave almost the same answer. In response to a question concerning whether the respondents experimented and explored welfare technology solutions in collaboration with potential end users such as clients/patients, the majority (184/336, 54.8%) of the female participants responded “never,” whereas 71 (21.1%) responded “yes, sometimes.” Among the male participants, 23/53 (43%) answered “never.” Respondents in patient-related professions, including the specialist dementia nurses and the chief responsible medical nurses, answered that they never experimented. The results also showed that professionals with longer work experience (more than 20 years) largely did not experiment and explore welfare technology with patients (91/140, 65.0%).

The next question examined whether the respondents experienced any problems in purchasing and exploring welfare technology in their profession/role (Multimedia Appendix 1, question 9). Both women (53%) and men (53%) reported experiencing such difficulties. The chi square test showed independence (*P*=.95) from gender; however, more than half the respondents indicated that they experienced problems. A clear association emerged with profession/role (*P*=.07). The majority of the IT personnel (19/29, 66%) answered that they had no such problems, whereas the majority of chief responsible medical nurses (41/78, 53%), OT/PTs (67/103, 65.0%), and specialist nurses in dementia care (26/51, 51%) did encounter such difficulties (see Table 2).

The next question asked whether the respondents regularly evaluated potential new welfare technology for their elder care organizations (Multimedia Appendix 1, question 10). These results showed independence of gender, age, and work experience. However, the majority of both the men (55%) and the women (62%) answered that they never evaluated new welfare technology. The results showed an association between profession/role and regular evaluation of new welfare technology (*P*=.02), with the majority reporting that they did not evaluate and test the suitability of new welfare technology (Table 3).

Responses to the question “Do you evaluate and follow up the welfare technology that is implemented?” were independent of gender. However, the association between profession/role and continuous evaluation of existing welfare technology was strong (*P*=.02), with the majority of participants answering that they do evaluate the welfare technology devices and solutions implemented in their organizations (Table 4).

**Table 2:** Distribution of responses regarding experimentation with and purchase of new welfare technology in relation to profession/role (N=393).

Profession/role	N	Yes, n (%)	No, n (%)
Information technology staff	29	10 (34.5 )	19 (65.5)
Chief medical nurse responsible	78	41 (52.3)	37 (47.4)
Chief rehabilitation officer responsible	28	14 (50.0)	14 (50.0)
Occupational therapist/physiotherapist	103	67 (65.0)	36 (35.0)
Specialist dementia nurse	51	26 (51.0)	25 (49.1)
Other (manager/electronic health strategist)	103	52 (50.5)	51 (50.1)

**Table 3:** Distribution of responses regarding continuous evaluation of potential welfare technology for future deployment in elder care in relation to profession/role (N=393).

Profession/role	N	Yes, n (%)	No, n (%)
Information technology staff	29	13 (44.8)	16 (55.2)
Chief medical nurse responsible	78	39 (50.0)	39 (50.0)
Chief rehabilitation officer responsible	28	11 (39.1)	17 (60.7)
Occupational therapist/physiotherapist	103	27 (26.2)	76 (73.8)
Specialist dementia nurse	51	17 (33.3)	34 (66.7)
Other (manager/electronic health strategist)	103	45 (43.7)	58 (56.3)

**Table 4:** Distribution of responses regarding continuous evaluation of implemented welfare technology in relation to profession/role (N=393).

Profession/role	N	Yes, n (%)	No, n (%)
Information technology staff	29	23 (79.3)	6 (20.7)
Chief medical nurse responsible	78	57 (73.1)	21 (26.9)
Chief rehabilitation officer responsible	28	22 (78.6 )	6 (21.4)
Occupational therapist/physiotherapist	103	57 (55.3)	46 (44.7)
Specialist dementia nurse	51	38 (74.5)	13 (25.5)
Other (manager/electronic health strategist)	103	75 (72.8)	28 (27.1)

### Procurement

The respondents’ involvement in the decision making related to buying and procuring welfare technology was also investigated (Multimedia Appendix 1, question 12). The chi square analysis showed that even though the majority of the participants were women, more men (30/53, 57%) were involved in the procurement process for welfare technology devices and solutions than women (98/336, 29.2%) (*P*<.001).

More than half of the chief responsible medical nurses (41/78, 53%) were involved in decision making related to buying and procurement, whereas the chief responsible rehabilitation officers (4/28, 14%), OT/PTs (7/103, 6.8%), and specialist nurses in dementia (5/51, 10%) reported little involvement. Most of the IT staff (27/29, 93%) were involved in welfare technology procurement decisions, but those who worked closely with the patients, such as the OTs (who prescribe most of the welfare technology), specialist dementia nurses, and chief rehabilitation officers responsible, had no such involvement.

## Discussion

### Principal Findings

The aim of this study was to explore and describe experiences of working with welfare technology in municipal elder care across gender, age, and profession. Some of the most interesting results are highlighted as follows. There was an overall positive attitude among the respondents toward digital transformation but the process was also perceived as too slow. Respondents are encouraged by management but never explore welfare technology with their managers. Moreover, elder care organizations are perceived to neither optimize nor experiment with new welfare technology. A significant decision-making and gender aspect was revealed, demonstrating issues with emancipation and welfare technology implementation. Finally, one of the key results from this study is that young personnel want to speed up the digital transformation.

Most of the respondents in this cross-sectional, explorative study were women, reflecting the nature of health care as a female-dominated occupation. In the gender distribution, the majority of IT staff and the group of “others” (eg, eHealth strategists and managers) were men. Women constituted the majority in the professions working closely with patients. The IT staff had less work experience (less than 4 years), whereas the other groups, especially the specialist dementia nurses, had far longer work experience. These demographic features of the sample are important to bear in mind to contextualize the discussion below.

### Positive But Slow Digital Transformation

In general, municipal elder care personnel viewed the deployment of new technology in their work positively. Society, media, and political discourse all express the expectation that welfare technologies offer potential solutions to the demographic challenges and shortages in health care staff, and that they could have positive effects on the personnel implementing the welfare technology [4,27]. In terms of the participants’ perceptions of the speed of digital transformation, the results showed that both men and women perceive the technological developments in elder care as being “too slow.” These results confirm the high expectations for welfare technology in society and demonstrate the reality that progress is very slow in everyday elder care practices.

### Personnel Are Encouraged to Explore and Experiment With Welfare Technology But Never Do

Other interesting results that emerged from the questionnaire concerned encouragement, experimentation, and exploration with welfare technology. The high level of encouragement from management that the different professional groups in municipal elder care experienced would seem to reflect the Swedish government’s positive views on the use of welfare technology [4]. This study shows that most management encouraged personnel to experiment with and explore welfare technology. However, most of the participants had never explored welfare technology with their managers. Based on these findings, it can be concluded that personnel (eg, eHealth strategist) make decisions on new technology purchases without management’s involvement in experimentation with the new welfare technology. Alternatively, it can be assumed that some welfare technology is procured and purchased without being tested. Further studies are needed to explore these aspects.

### Elder Care Personnel Neither Optimize Nor Experiment With New Welfare Technology

Participants gave negative responses as to whether they perceived their workplace as optimizing the use of welfare technology. Those working closely with patients, including OT/PTs and specialist dementia nurses, perceived that the elder care organizations could optimize the use of welfare technology to a greater extent. However, some of the participants also responded that technology had changed health care organizations in negative ways. These findings might indicate a negative reaction to the rapid technological change in society or the overburdening of these professions/roles with responsibilities in their everyday work.

The association of profession/role and experimentation with new welfare technology was strong, with a majority of the respondents answering that they never experimented. Interestingly, the participants felt encouraged to experiment with and explore welfare technology but never did so with either management or patients. These findings could indicate that patients in need of elder care have little influence on the kinds of welfare technology recommended and implemented. Substantial research has shown the importance of patient participation in the procurement process for assistive and welfare technologies [28,29-33]. Evidence supports the conclusion that patients use assistive technology and welfare technology more if they are involved in the entire process of its procurement [34].

### Decision Making and Gender

More than half the female participants reported that they “use technology when most people do,” whereas the majority of men responded that they “like new technology and use technology before most people.” These findings support earlier research showing that men have greater confidence in using new technologies than women [35]. Studies on gender and technology have shown that the introduction of new technologies tends to degrade women’s work and separate it from men’s work [36], while women tend to be replaced by men during technological change in the workplace [37]. As welfare technology enters the generally female domain of elder care, men seem to carry the professional role (IT strategist) of introducing the welfare technology into municipal elder care. The present results showed that men have a more positive attitude toward and curiosity regarding new technologies than women. This may result from their role in the organization and may not be due to a gender effect itself. However, this finding raises the question as to whether the introduction of technology into municipal elder care will lead to an increase in male care professionals.

Considering welfare technology procurement, the results showed that although most of the participants were women, more male respondents were involved in the decision making than women. These results confirm that technology is often constructed as a male domain [38,39], and that it is not neutral but is rather shaped by social values and norms [40,41]. These results indicate that the male respondents had more power than the female respondents in the decision-making process regarding the deployment and use of technological innovations in the elder care sector.

Historically, women have experienced the negative effects of hierarchical structures of power and technology deployment [37,38,41-43]. The digital transformation has, and will continue to have, major impacts on many aspects of everyday and professional life, including gender equality. Digital transformation will affect political participation, the nature of the labor market, and interactions with friends and colleagues, opening opportunities for dealing with gender differences that affect both women and men, albeit often differently [43]. Digital transformation has the potential to either reproduce and maintain gender expectations and power structures or to advance a more equal society. Digitization and technological innovations take place not in a vacuum but in interactions with social, cultural, economic, and political factors [44].

Personnel in municipal elder care are shaped by their working conditions, and, unfortunately, our results indicate that welfare technology maintains and reproduces gender expectations and power structures in municipal elder care. For example, the male participants had much higher levels of power in the decision-making process by which new technological solutions were selected for implementation in municipal elder care.

### Emancipation and Welfare Technology Implementation

Almost all of the IT staff and the chief rehabilitation officers responsible were involved in procurement decisions, but among those working closely with patients, OT/PTs and specialist dementia nurses did not participate in these decisions. These results suggest that those with strong power in the procurement process do not work closely with patients or even regularly meet them, while those who work directly with patients daily and implement the new technological solutions have little power and do not control what solutions or devices are procured. The consequence might be a resistance to technology among frontline care personnel and patients in municipal elder care [10,15,45,46]. Recent studies have analyzed aspects of participation among different care professionals when welfare technologies have been implemented [12,17,19,20,45-49]. These studies have shown that the work of the individual care personnel is crucial in technology deployment in elder care and that technology innovation also changes the dynamics within practices [21]. However, this research has generally failed to acknowledge the participation or lack of participation by care professionals in the initial exploration and experimentation with welfare technology before procurement and implementation, which requires further study. Research on well-being in the workplace has shown that having control and a voice in the workplace are key factors for well-being and satisfaction at work [50-53]. A lack of a voice on which welfare technology solutions are implemented may therefore negatively affect those working closely with patients, consequently increasing their work-related stress and administrative load [54].

### Young Personnel Want to Speed Up the Digital Transformation

Our findings indicate that not only gender but also age affect the perception of welfare technology. The association between age and perceptions of the speed of digital transformation shows the importance of age as a variable. All of the 18 to 24-year-old participants thought that the speed was “too slow,” whereas more than half of the 65 to 74-year-old participants thought that it was moving at the “right pace.” These findings indicate that the perception that digital transformation is proceeding at the right pace increases with age. Prensky [52] defines “digital natives” as those who have used and been surrounded by technology their whole lives and “digital immigrants” as those who learn and adopt to technology and have had experience of doing similar tasks without technology. Prensky [52] proposed that digital natives have significant advantages when using technology. This was confirmed by our study results in which the younger respondents declared that they “like new technology and use it before most people do.”

### Limitations

One limitation of this study was that the questionnaire was sent to municipal registrars who passed it on to potential participants working with welfare technology, resulting in the participants occupying different levels and roles in organizations, and included registered nurses, OT/PTs, managers, and IT strategists. It would have been interesting to determine the level at which the individual participants worked in terms of how much decision-making power they had for buying and implementing welfare technology; however, this was not the aim of the study. Another limitation was that the questionnaire was anonymous, and therefore the geographic spread of the participants in Sweden was unclear. However, a total of 393 responses were received and Sweden has 290 municipalities, suggesting a good geographic spread.

### Conclusion

The findings show that various professions working in municipal elder care hold very positive views on new technologies. However, there are both gender and age differences in attitudes toward technology (for the use of technology in general and in the workplace) and in participation in decision making for the procurement of new welfare technologies. The consequences of this might include personnel’s resistance to welfare technology in municipal health care [46], which would affect clients and their potential participation in welfare technology. Therefore, the people working closely with patients who are expected to implement the new technologies need to be more involved in the procurement process.

Among other findings, a majority of the IT staff were men who had worked in municipal elder care for less than 4 years, and most of these participants were involved in welfare technology procurement decisions. These findings imply that men with little work experience or knowledge of elder care carry high levels of power and responsibility for decisions that will be executed by the female-dominated groups of elder care personnel. The decisions of these few could have a major influence on future elder care as digital transformation changes how work is carried out in municipal elder care.

## Appendix

Multimedia Appendix 1 Questionnaire on ongoing digital transformation in municipal elder care. WT: welfare technology.

Multimedia Appendix 2 Associations among variables for analysis. WT: welfare technology.

## Abbreviations

eHealth: electronic health
IT: information technology
OT: occupational therapist
PT: physiotherapist
