# Supplementary material for: Views of Swedish Elder Care Personnel on Ongoing Digital Transformation: Cross-Sectional Study
Source: J Med Internet Res. 2020 Jun 16;22(6):e15450. doi: 10.2196/15450 (PMC7327600; doi:10.2196/15450)
Supplement: Multimedia Appendix 1 [file jmir_v22i6e15450_app1.docx]

**MULTIMEDIA APPENDIX 1.**

Questions from questionnaire; Ongoing digital transformation in municipal elder care.

### Perceptions of the Speed of Change

1. Grade the speed of the digital transformation in your workplace
2. What do you think about this statement: technology and digitalization have made health care organizations change too fast?
3. Does this statement confirm your perceptions of the deployment of WT in your municipal elder care organization: my workplace utilizes WT optimally?
4. Regarding technology, what describes you best?

### Encouragement, Exploration, and Experimentation with Welfare Technology

1. Does the management of your elder care organization or your closest superior encourage you to use new WT solutions?
2. Do you experiment with new WT in your everyday work?
3. Do you explore and experiment with new WT in collaboration with the management of your elder care organization or with your closest superior?
4. Do you explore and experiment with new WT in collaboration with potential end users such as patients?
5. Do you perceive any problems when you want to explore and buy new WT solutions?
6. To what extent do you evaluate whether potential WT fit into your organizational context?
7. Do you evaluate and follow up the WT that is implemented?

### Procurement

1. Are you involved in the WT procurement decisions?
