# Supplementary material for: Views of Swedish Elder Care Personnel on Ongoing Digital Transformation: Cross-Sectional Study
Source: J Med Internet Res. 2020 Jun 16;22(6):e15450. doi: 10.2196/15450 (PMC7327600; doi:10.2196/15450)
Supplement: Multimedia Appendix 2 [file jmir_v22i6e15450_app2.docx]

**APPENDIX 2**

**Chi Square Tests of the Correlations of the Variables**

**Perceptions of Speed of Change**

- Correlation of grading the speed of the digital transformation of the workplace with gender, age, work experience, and profession/roles
- Correlation of too rapid changes in health care organizations due to technology and digitalization with gender, age, work experience, and profession/roles
- Correlation of personal perceptions of how the workplace optimizes WT with gender, age, work experience, and profession/roles
- Correlation of technology that describes one with gender, age, work experience, and profession/roles

**Encouragement, Exploration, and Experimentation with WT Solutions**

- Correlation of encouragement from management to use of WT in everyday work with gender, age, work experience, and profession/roles
- Correlation of experimentation with new WT with gender, age, work experience, and profession/roles
- Correlation of experimentation and exploration of WT with management with gender, age, work experience, and profession/roles
- Correlation of experimentation and exploration of WT with clients/patients with gender, age, work experience, and profession/roles
- Correlation of experimentation with and purchases of new WT with gender, age, work experience, and profession/roles
- Correlation of continuous evaluation of potential WT for future deployment in elder care organizations with gender, age, work experience, and profession/roles
- Correlation of continuous evaluation of implemented WT with gender, age, work experience, and profession/roles

**Procurement**

- Correlation of involvement in decision-making for the procurement of WT with gender, age, work experience, and profession/roles
